# Critical reflections, challenges and solutions for migrant and refugee health: 2nd M8 Alliance Expert Meeting

**DOI:** 10.1186/s40985-019-0113-3

**Published:** 2019-03-19

**Authors:** Nefti-Eboni Bempong, Danny Sheath, Joachim Seybold, Antoine Flahault, Anneliese Depoux, Luciano Saso

**Affiliations:** 1 Institute of Global Health, Faculty of Medicine, University of Geneva, Geneva, Switzerland; 2 Charité – Universitätsmedizin Berlin, Berlin, Germany; 3 Centre Virchow-Villermé for Public Health Paris-Berlin–Paris Office, Paris, France; 4 Faculty of Pharmacy and Medicine, Sapienza University of Rome, Rome, Italy

**Keywords:** Migrant health, Refugee health, Health policy, M8 Alliance, Tuberculosis, Mental health, Screening

## Abstract

Throughout recent years, we have witnessed an increase in human migration as a result of conflict, political instability and changes in the climate. Despite the growing number of migrants and refugees, provisions to address their health needs remain inadequate and often unmet. Whilst a variety of instruments exist to assert and emphasise the importance for migrant and refugee health, the lack of shared priorities between partners and stakeholders results in poor access to healthcare and essential medicines.

In response to the growing health challenges faced by migrants and refugees, members of the *M8 Alliance* launched an annual *Expert Meeting on Migrants*’ *and Refugees*’ *Health*. This report is shaped by discussions from the second M8 Alliance Expert Meeting (Sapienza University of Rome, Italy, 15–16 June 2018) and is supported by supplementing literature to develop a framework addressing critical reflections, challenges and solutions of and for migrant and refugee health. This report aims to inform decision-making fostering a humanitarian, ethics and rights-based approach. Through a series of country-specific case studies and discussions, this report captures the most prominent themes and recommendations such as mental health, tuberculosis (TB) and best practices for increased access.

## Narrative of migration: Moving lives

As a complex and social phenomenon, the process of migration has become increasingly political, with adequate healthcare often low on the list of priorities. Over the past decade, there has been an influx of migrants crossing borders, primarily due to political instability, military conflict and extreme climatic conditions. These events have been accompanied by a growing burden of disease, with data suggesting that infectious disease, accidents, injuries, musculoskeletal disorders and violence disproportionately affect migrant groups compared to long-settled populations in the European Union [1]. Amongst these health challenges, mental health disorders and TB remain a major problem. Disease prevalence varies between migrant groups, and therefore it is important to be aware of the different types of migrants that exist [1].

Whilst a refugee is a type of migrant, stark differences exist between refugees and other type of migrants. The *Convention and Protocol relating to the status of Refugees* defined refugees as “Individuals who, owing to a well-founded fear of being persecuted for reasons of race, religion, nationality, membership of a particular social group, or political opinion, are outside the country of their nationality, and are unable to, or owing to such fear, are unwilling to avail themselves of the protection of that country or return because of fear of persecution” [2]. Other migrant status may include international migrants, internal migrants, irregular migrants and tourists [3]. The main difference that exists between refugee populations (asylum seekers, resettled and relocated refugees) and other types of migrant groups is that refugees are survivors of persecution and multiple violent events, including war and torture, and their migration experience is forced [4]. The differences in these lived experiences may therefore have a profound effect on their overall wellbeing and in particular on their mental health [4]. In addition to categorising the type of migrant, the process of migration may also be categorized. Factors that dictate the type of migration include the following: boundary crossed (national, international, political, and administrative), duration of stay (temporary, permanent), distance (regional, national, and international) and lastly, the decision-making approach for migration [5]. The latter can be further subcategorised into voluntary, instigated or forced, or impelled [5]. Migration, both voluntary and forced, is increasing at an unprecedented rate, leaving many unanswered questions for public health. Therefore, migrant and refugee health requires a collective response, addressing health challenges by mobilizing stakeholders, trade unions and partners globally.

**Fig. 1:**
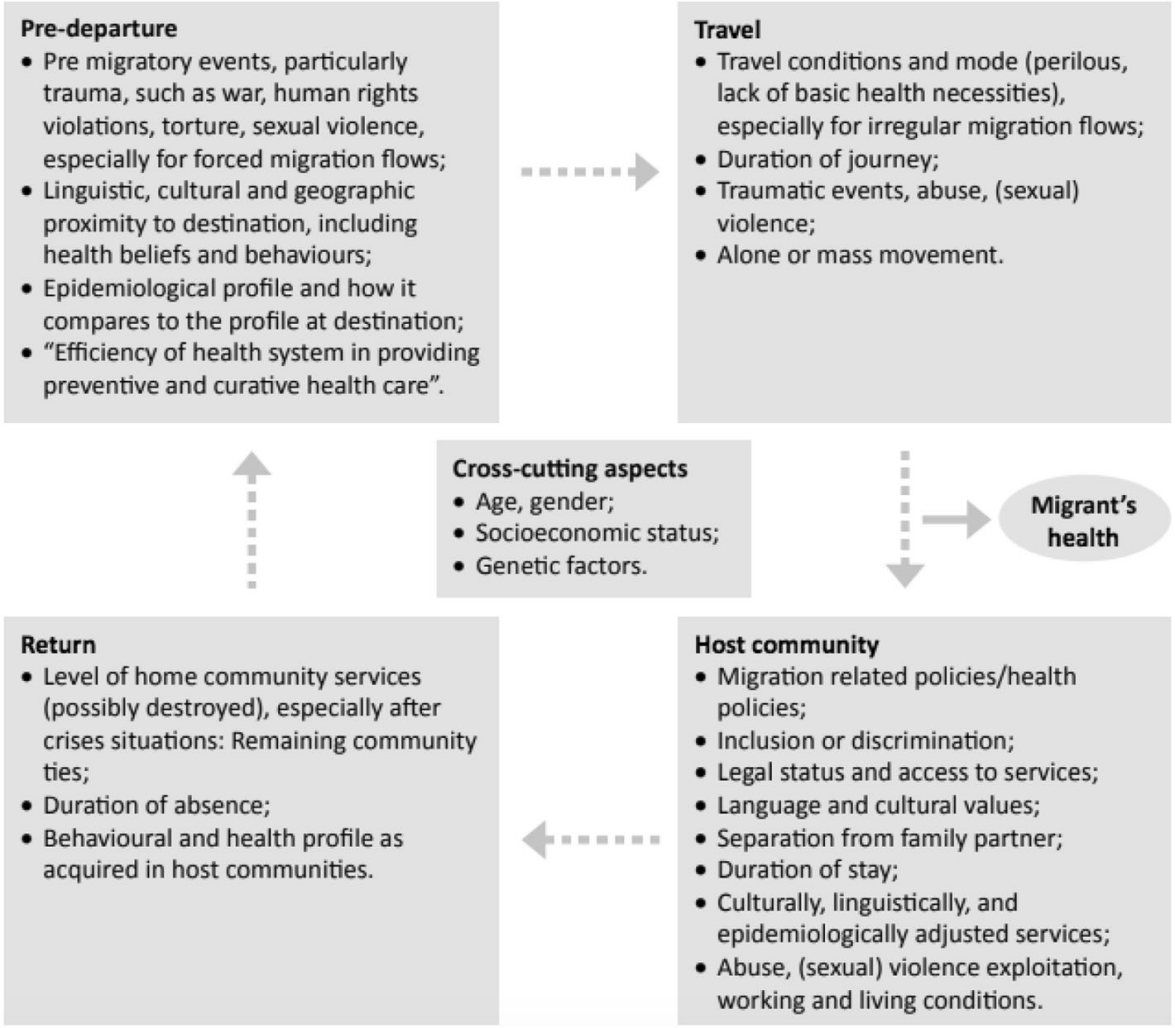
Aspects of the various migrant stages that can affect migrants’ health [9]

The migration process can increase migrant and refugees vulnerability to ill health, through increased exposure to risk factors (see Fig. 1). The process may be subcategorised into the following: pre-departure and at the border, travel and transit, host communities and return. Compulsory medical screening is often a major concern for migrants and refugees pre-departure or at the border, as unsuccessful screening could result in denial to enter their chosen host country. The purpose of screening is to address the introduction of potential health threats that may endanger the health of host populations, specifically for the case of infectious disease [6, 7]. However, the legitimacy of medical screening has been questioned, as it disregards the moral and ethical implications, and also does not adequately address diseases with latent periods [8]. The migratory journey itself affects the health of many migrants, due to physical and environmental threats, the lack of access to the most basic services, alongside increased exposure to both violence and trauma having significant repercussions on their mental health [9].

Once within the host country, there are still many obstacles hindering health for migrant populations, such as occupational health and safety. Migrants and refugees are more commonly exposed to occupational hazards via physical labour in occupations such as mining, agriculture and construction, whilst also having increased exposure to sexual exploitation [10]. Upon return to their country of origin, health problems that have been acquired in the host country may surface—this is especially true for mental health conditions, which may increase in severity [11].

### Understanding the issue

The portrayal of migration in the media often leads to false beliefs, stereotypes, and negative perceptions of migrants and refugees. This widely circulating narrative often dilutes the severity of the phenomena, focusing only on negative aspects and shedding no light on the positives of migration. As a result, host societies often neglect to understand migration in its entirety (from departure to arrival and integration), especially within its political framework [1]. The *Wroclaw Medical University*, *Poland*, further explored public opinion regarding migration by *Attitudes and opinions of Polish society about immigrants and refugees in Poland* [12]. Results were drawn from a survey on public acceptance of refugees and migrants, including 367 respondents (of which 100 were within medical professions), conducted in cooperation with the Polish Association of Healthcare Managers (STOMOZ). Results of the survey seemed contradictory at times, indicating that many respondents perhaps misunderstood or misinterpreted the concept of migration. For instance, when asked if “every person should be able to move to another country fleeing war or prosecution” 62% of respondents answered “totally agree”, however when asked “would you feel uncomfortable if your new neighbour was an immigrant of refugee”, 54% of respondents answered “yes” [12]. This indicated that whilst respondents seemed to understand the reason for leaving, they were less receptive to the practical realities of migration.

Findings from the survey concluded that a possible reason for the negative opinions could be deduced to (1) misunderstanding of migration, (2) fear of an unknown situation or (3) stereotypes [12]. However, it is also important to note that the migrant population in Poland consists mostly of Ukrainian immigrants, who due to proximity have acclimatized well in Polish society. Similarities within the neighbouring regions, as well similarities in ethnicity, may have therefore accounted for some of the discrepancy between responses in this case [12]. Additionally, it is important to note that a rather small sample size of the Polish society were included in the survey and are thus not representative of the Polish society as a whole.

### Evidence-based narrative on migration to promote reliability, coherence and consistency

In order to move beyond simply understanding migration and to better foster admission and integration, the narrative of migration needs to be transformed by evidence-based, reliable and sustainable data sets. A primary example in which migrant groups are often misrepresented is with regard to economic resources. Migrants are often perceived as a long-term drain on the economy, when in reality it is a short-term cost for long-term benefits. For example, whilst initial absorption may be costly, it has been estimated that in the UK, refugees are projected to grow the GDP more than two-fold (€126.6 bn) in the next 5 years, through the creation of jobs, increased demand for services and products, and also filling gaps in EU workforces [13]. Additionally, findings from *Addressing the income gap of ethnic groups*: *impact of health on livelihoods* conducted by the *University of Economics in Bratislava* demonstrated that proxies for migrant discrimination played a substantial role in explaining the poverty differences between migrants and nationals in the EU. Therefore, it remains imperative to change the narrative of migrants through the use of data, to support and promote the evidence on the positive effects of migration.

The inclusion of evidence-based tools also needs to be reinforced and adopted at country-level. In Italy, two guidelines, namely the *Guidelines to control TB among migrants in Italy* and *Boarder checks kept in check*, were developed in collaboration with the National Institute of Health, the National Institute for Health, Migration and Poverty, and the Italian Society of Migration Medicine to promote evidence-based guidance for decision-makers [14]. The guidelines were drawn from systematic and rigorous literature reviews, which aimed to draw recommendations and best practices focused primarily on infectious diseases, chronic-degenerative conditions, pregnancy and vaccinations [14]. Rewriting the true narrative of migration should be based on both values and human rights, in hopes of triggering further engagement and acceptance. Similarly, shaping perception is also part of the Global Compact, which as part of its framework, aims to “eliminate all forms of discrimination and promote evidence-based public disclosure to shape perceptions of migration” [15].

### International frameworks and policy: Translating abstract concepts into sustainable action

Due to the political nature of migration and the implications for the economic and legal system, it remains essential that there are just and robust frameworks and policy to advocate for the human rights of migrant populations. In collaboration with the WHO framework of priorities and guiding principles to promote health of refugees and migrants, the Global Compact for Migration seeks to set out comprehensive and holistic guidelines for healthier lives of migrant and refugees [15]. Objective 15f in the Global Compact for Migration seeks to provide access to basic services for migrants and states the following:

Expand and enhance national health systems, incorporating the needs of migrants in national and local health care policies and plans, including by strengthening capacities for service provision, facilitating affordable and non-discriminatory access, reducing communication barriers, and training health care providers on culturally-sensitive service delivery, in order to promote physical and mental health of migrants and communities overall.

**Fig. 2:**
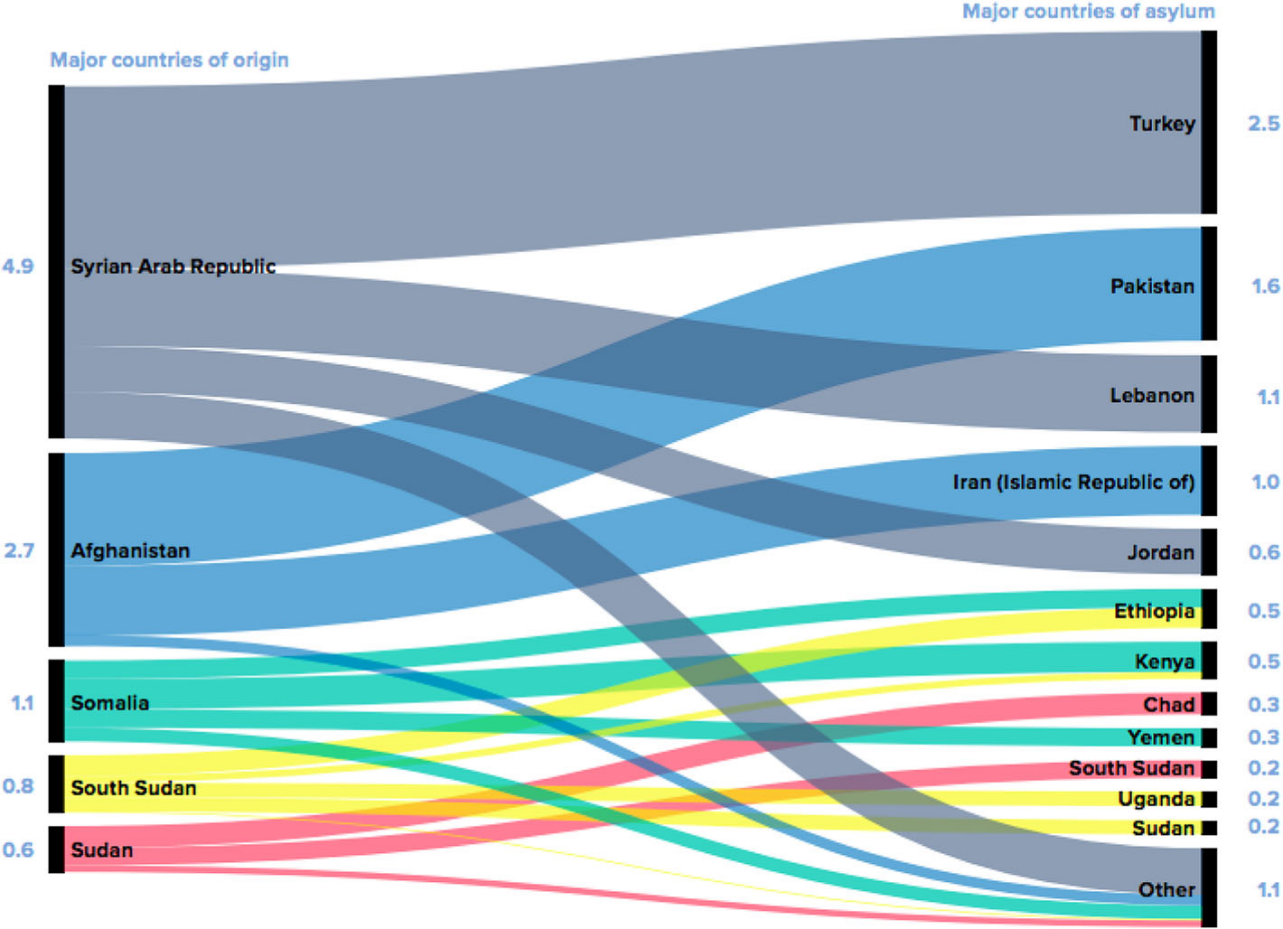
Where refugees from the top five countries of origin found asylum (UNHCR, 2015)

There is a clear link which exists between migration and development, not only demonstrated by the Global Compact acting as a response to the sustainable development goals, but also by the proliferation of universal health care for migrants and refugees in Iran. There has been an influx of Afghan refugees into Iran, which currently hosts 951,142 documented refugees and is estimated to also host 1.5 to 2 million undocumented refugees (see Fig. 2) [16]. In the case for documented refugees, insurance coverage for refugees is endorsed by Iran’s development programs, namely through the 6-year development plan (2016–2021; Article 70, number 5). Examples of free primary healthcare services in Iran include vaccinations, maternal and child health, family planning and psychological consultations [17]. Health is also a main pillar of the United Nations Sustainable Development Goals, as a healthier population will also reinforce greater financial sustainability.

According to Lee’s push and pull theory, there may be various push and pull factors which influence migrants’ choice of host destination, and access to medicines may be categorised as a pull factor to migrate to said host country [18]. However, pull factors may be extremely conditional at times, widening the gap between expectation and reality. For instance, in France, a health protection scheme for undocumented migrants was created in 2000, named the State Medical Assistance [19]. Whilst the scheme had a period of entitlement for up to one renewable year, the legitimacy of assistance for undocumented migrants has been subject to debate and regular criticisms. The reasons for this being the requirements needed to access the healthcare are not only were undocumented migrants expected to have lived in the territory for over 3 months, but also beneficiaries were expected to bring forward supporting documents such as proof of identity, address of domiciliation and administrative documents proving their 3-month presence in France [19]. These criteria increase the difficulty to actually access these services, with only 10.2% of eligible candidates gaining effective access to medicine and primary health care services [19]. This echoes the need to have clearer laws and regulations, and research also concluded that it might be more beneficial to reintroduce undocumented migrants into a common law system opposed to maintaining a specific defined system for undocumented migrants [19].

## Mental health

Mental health remains a major challenge for migrant and refugee health, with a notable increase in schizophrenia, depression and anxiety. Due to the stress-inducing nature of migrations, difficulties may arise as a result of poor social skills, the concept of self and further exposure to psychological, social and biological vulnerabilities [20]. Mental health may afflict both children and adults, with exposure to violence, internalising difficulties and unaccompanied transit identified as the key risk factors for poor mental health in children [21]. Poor social interaction and integration have been hypothesised as key factors affecting mental health adjustment in adults [20]. Cultural shock plays a major role in reduced acclimatisation and has been hypothesised to occur in six stages: strain; sense of loss and feelings of deprivation; rejection by and members of the new culture; confusion in role and role expectation, values, feelings and self-identity; surprise, anxiety, disgust and indignation; and feelings of impotence [22]. Furthermore, the novel environment with uncertainties potentially caused by residence status, limited access to basic infrastructures and health services due to living conditions in provisional refugee shelters can cause emotional distress likely to increase prevalence rates of mental disorders amongst refugees in comparison to the general population [23–25]. Post-traumatic stress disorder (PTSD) has been observed in most migrant and refugee populations, due to the heterogenic nature of the migration process itself [20].

Trauma is especially prominent in refugee populations, often categorised as either “collective traumas”, which refer to shared injuries to a population’s social, cultural and physical ecologies [26], or “social suffering” described as “interconnected adversities on the level of individual, family, community and society” [27]. It is important to note that trauma may manifest pre-migration, but also post-migration and post-displacement adjustment [28]. Displacement and pre-migration situations of war and conflict may involve: witnessing or being subjected to torture, killings, atrocities, incarceration, starvation/deprivation, rape, sexual assault and physical beatings [28]. Trauma often results in PTSD, and the four main resettlement stressors predictive of PTSD have been identified as (1) social and economic strain, (2) loss of status corresponding with racism and discrimination, (3) threats and violence and (4) alienation [28]. However, exposure to other stressful and traumatising experiences may also contribute, such as abuse by law enforcement officers, separation from families and fear of detention and/or deportation. Trauma has also been associated with major depressive disorders and suicide [29]. To start healing from traumatic events, it requires refugees, both individually and collectively, to make meaning of their trauma [4, 30].

**Fig. 3:**
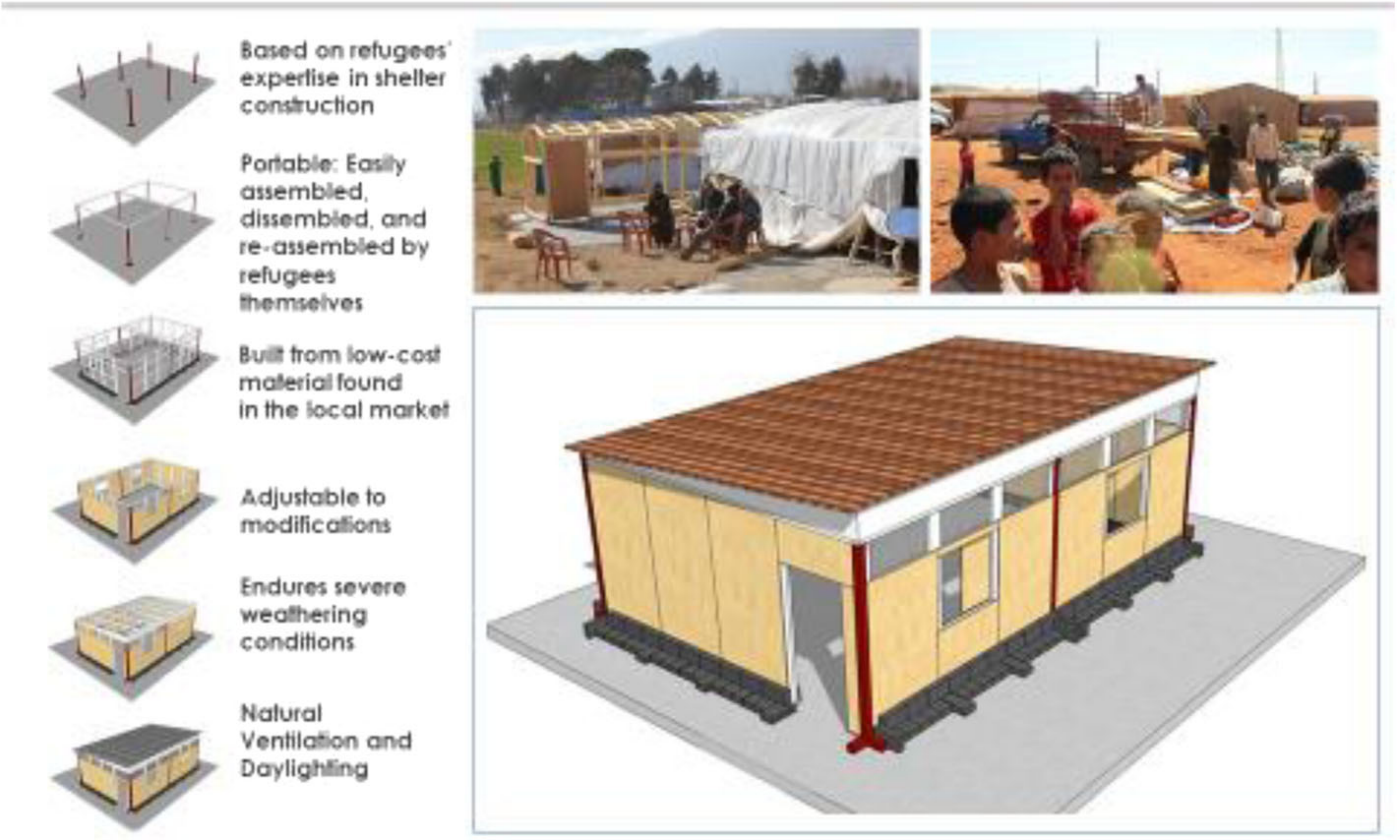
Ghata school unit [31]

More recently, the *Centre for Civic Engagement and Community Service*, *at the American University of Beirut* (*AUB*) launched the *Ghata school* project in Lebanon [31]. The aim of these schools was to produce a restorative built environment to impact refugee’s mental health, promoting the notion of a “safe place” via the following objectives: (1) to assess the frequency of mental health problems and trauma outcomes (PTSD, depression, anxiety) amongst children aged 12–14 attending Ghata versus tented schools; (2) to assess the perception of children and parents on their schools as a restorative built environment; and lastly, (3) to assess the need for mental health service interventions within the built environment [31]. The design of the Ghata unit is derived from refugees’ own shelter construction practices that are based on simplicity, portability, adaptability, scalability, climatic responsiveness and economic efficiency (see Fig. 3) [31]. AUB student volunteers assembled the first prototype in 2013, and two refugees built the second in six working hours; specifically choosing land in close proximity of refugee resettlements. Following rigorous simulation analysis, the project was scaled up across the country with support from the Ministry of Education. To date, 10 portable school campuses located within refugees’ informal tented settlements have been assembled, serving around 5000 students annually—with attendance rates over 80% [31, 32].

Results from the MHPSS surveys allowed informants to learn more about their experiences and trauma. For instance, 59.3% described their journey as “terrifying and scary”, whilst 35.6% shared their experience of seeing someone being “beaten up, shot or killed by another person”, and 45.8% admitting that they “felt like they were going to die” [31]. Ghata aims to fill the gap for needs and resource assessment, and it has also been noted that education can be used as an instrument to foster social integration, by increasing engagement and acceptance within resettled communities. Mental health within migrant communities remains a major challenge, and therefore it is important to also immobilise stakeholders and trade unions for an interagency approach. It is important to not only address the fear, anxiety and doubts pre-migration, but also assess mental health needs post-migration, guaranteeing cohesion of migrant communities.

## Tuberculosis

Tuberculosis (TB) is an airborne infectious disease caused by the bacterium *Mycobacterium tuberculosis*, which affects the lungs most commonly, and is spread from person to person by coughing and sneezing [33]. TB is entirely preventable and treatable yet it is one of the top 10 causes of death globally, responsible for the deaths of 1.7 million in 2016, with most of these (over 95%) deaths occurring in low- and middle-income countries [34]. Clearly, TB disproportionally affects poor and vulnerable populations hence migrant populations are at a heightened risk of contracting the infection. Further, migration increases TB-related morbidity and mortality due to the conditions they are exposed to during migration itself including malnutrition, overcrowding in camps and treatment interruption, which can increase the risk of drug resistance [35]. Even when migrants reach their final destination countries, their TB-associated morbidity and mortality is elevated. This is particularly true for undocumented migrants where fear of deportation or detention prevents individuals seeking medical attention for diagnosis or treatment. Those that do identify themselves may end up in detention centres where conditions are often crowded, proliferating the spread of infection and disrupting access to treatment. The situation is not much better for migrants with legal status, whose access to care and treatment is often reliant on their working permits and health insurance, exposing a significant proportion of this group to inadequate services [35]. As an example, in Germany, the highest incidence of TB (18.2/100,000) was found in young adults aged 20 to 24 years (male 25.9 vs. female 9.7). Furthermore, TB was diagnosed significantly higher in the foreign population at all ages (42.6/100,000) compared to the general population (2.2/100,000) [36]. Therefore, all screening activities for tuberculosis should take into account that particularly migrated adolescents and young adults are at a much higher risk to be diagnosed with TB.

A particular migrant subgroup that requires attention is the unaccompanied asylum-seeking minors (UASM). UASM are defined as “third-country nationals or stateless persons below the age of 18, who arrive on the territory of the Member States unaccompanied by an adult responsible for them whether by law or custom, and for as long as they are not effectively taken into the care of such a person; it includes minors who are left unaccompanied after they have entered the territory of the Member States” [37]. Given the scale of migration amongst this vulnerable subgroup, it is important that they are not missed from screening and treatment programs.

At the 2014 World Health Assembly, the WHO End TB Strategy was introduced as a blueprint for countries to end the TB epidemic by reducing TB deaths and incidence and eliminating the terrible costs of the epidemic. The End TB Strategy outlines global impact targets to reduce TB deaths by 90%, to cut new cases by 80% between 2015 and 2030, and to ensure that no family is burdened with catastrophic costs due to TB [38]. This goal of 2030 is shaped somewhat by the inclusion of the End TB strategy in the health targets of the Sustainable Development Goals (SDGs). WHO has gone a step further than this by setting the target of 95% reduction in deaths and a 90% decline in TB incidence globally by 2035; this is similar to current levels in low-TB incidence countries today and represents a commitment to bring high-burden countries in line with this.

The End TB Strategy faces a number of challenges on the path to achieving these goals, including the growing threat of multidrug resistance, but the status of TB amongst the migrant populations also represents a key challenge. In fact, these two challenges are not mutually exclusive, and migrant populations can accelerate the prevalence and spread of multidrug resistant TB as seen with the arrival of new strains of drug-resistant TB in the Western Cape province in South Africa, thought to be a result of the large migrant population [39]. Such introductions of new resistant strains of TB emphasise the need for the inclusion of strain data in screening and notification systems within countries with large migrant populations, when there are sufficient resources available [39].

A key component of the primary pillar for the End TB Strategy is systematic screening of contacts and high-risk groups, one such high-risk group is migrant populations, hence the importance of clear and effective screening procedures in the context of migrant and refugee health. Screening is defined as “a process of identifying apparently healthy people who may be at increased risk of a disease or condition. They can then be offered information, further tests and appropriate treatment to reduce their risk and/or any complications arising from the disease or condition” (UK National Screening Committee, 2012). Healthcare workers who are in contact with a person coming from a TB high-prevalence country (> 100/100.000) should collect TB history and check for any TB symptoms or signs as standard practice. If TB is suspected, full diagnostic exams should be carried out immediately, and full preventive therapy must be offered. As previously mentioned, there are challenges unique to the migrant population, particularly regarding unregistered migrants, and these permeate down to make effective and comprehensive screening difficult. UASM present a special case, where a more general medical screening is conducted, with more in-depth examinations carried out (e.g. X-rays) in cases of risk of TB exposure or suspected infection.

Adherence remains a challenge at all stages from comprehensive screening procedures to the completion of preventative and treatment courses. Adherence is favoured through integrated management of cases, but many challenges to achieving this exist. Preventive therapy is usually well accepted amongst migrants, but major obstacles that derive from a lack of information or bad communication and organisational problems still persist. For example, in Italy more than 40% of screened patients testing positive for possible TB infection fail to undergo follow-up diagnoses. Around 25% of those patients that do undergo diagnostic X-rays and test negative choose not to use preventive prophylaxis, and of those that do 64% still fail to complete the course. That said, such problems have started to be overcome in recent years, and whilst a 64% failure to complete figure is still too high, this is a marked improvement over recent years.

## Challenges

Whilst migration can have positive impacts—particularly for development, there are also a number of challenges that exist as a by-product of increased migration. A major problem is the effect of increased workload on humanitarian and health workers. Greece has experienced many health workers prone to burn out, resulting in the development of a brain drain culture (the emigration of highly trained or skilled persons). For instance, a thematic analysis “*Understanding healthcare access for refugees in Greece*” conducted by the *Imperial College London* highlighted how socio-cultural differences such as language acted as a major barrier in communicating. The study also noted a low presence of translators exacerbating the negative consequences. Within the Greek experience, in addition to inadequate staffing, the lack of coordination and changes in available healthcare provision also contributed to the burn out of healthcare workers [40]. The language barrier may also contribute to the lack of adherence to treatment, delays or misdiagnosis, unnecessary examinations and incorrect treatments [9]. Linguistic problems are closely tied with cultural issues. More recently, the need for “culturally competent” conduct has been pushed on the healthcare agenda, which in the case of migrant health, means putting aside personal biases and being familiar with the health, social, cultural, religious and gender-related issues regarding the experience of migrant populations [41].

Another persisting problem that has been noted is the increased incidence of work-related injuries—the ILO estimated that there were 2.3 million occupational fatalities from a variety of sources in 2014, globally [42]. The department of public health and infectious diseases at the *Sapienza University of Rome* concluded in their study *Work-related injuries and mortality among immigrant workers in Italy*: *a national perspective*, that occupational injuries have more frequently been observed in migrant populations with increased hazardous exposure, which may include physical, chemical, biological or increased levels of psychosocial stress. Migrants are often engaged in what is known as 3-D jobs, dirty, dangerous and demanding, in which they experience a combination of increased workplace demands, lack of safety standards and frequent workplace abuse [42].

Writing a true narrative of the migration experience remains a challenge, as the narrative of migration needs to be evidence based in order to promote a future in which we build with trust. Amongst the need to mobilise stakeholders and trade unions, shape perception via media outlets, and enhance international cooperation, many other challenges exist. It is therefore crucial that migrant and refugee health continues to be addressed, through shared priorities and the vision of involved partners and stakeholders. With a changing landscape in the climate and demographics, unpredictable political outcomes and the growing emergence of infectious disease outbreaks, migration will continue to occur, and therefore advocating for health for all and issuing calls for action remain of up most importance.

“Ultimately, the challenge is to make migration work for all.”

-Antonio Vitorino, IOM Director General

## Recommendations

The symposium highlighted several critical areas for action, including the following:

- There is a need for an *evidence-based narrative* on migration to promote reliability, coherence and consistency.
- Migrant and refugee health must become a *shared priority* mirrored in the commitment of member states to ensure access to healthcare for all.
- The need to raise awareness of migrant group barriers to accessing care, and *training health care professionals* to overcome language barriers
- Support *social integration* through education, housing and employment
